# Audit of telephone HIV clinics: effective and acceptable

**DOI:** 10.1186/1758-2652-13-S4-P170

**Published:** 2010-11-08

**Authors:** WFC Woolley, C Babu, A Sukthankar, O McQuillan

**Affiliations:** 1 Manchester Royal Infirmary, Manchester Centre For Sexual Health, Manchester, UK

## Purpose of the study

A Telephone HIV clinic was set up and offered to patients with chronic, stable HIV-infection to replace face-to-face appointments for three out of their four HIV reviews in a 12 month period. We initiated these telephone clinics in 2007. We audited the effectiveness and acceptability of these Clinics.

## Methods

A total of 72 patients were included in the audit: 88% were male, 68% were men who have sex with men, 22% were heterosexuals of non-UK origin. This population reflects the wider patient cohort of this busy, city centre Genitourinary Medicine clinic. A retrospective case note analysis was conducted of patients seen in the telephone clinic over an 8 month period from December 2008 until July 2009. Clinic eligibility, consent documentation, compliance with recommended frequency of appointments and default rates were assessed. A patient satisfaction survey was conducted during this period.

## Summary of results

Eligibility for the telephone clinic was met in 77% of cases. In the rest (23%), failure to meet inclusion criteria related to the limit set for CD4 count (N=7), viral load (N=1), previous poor appointment attendance (N=5) and outstanding contact tracing issues (N=1). Consent was documented in 82% of cases. 94% of telephone appointments were conducted within the designated time frame and the correct ratio of telephone to face-to-face appointments was achieved in 63% of instances. Overall attendance was better for the telephone clinics with only 15% of appointments failing to make contact as compared to 23% DNA rates for the equivalent face-to-face clinics. There were no cases where an urgent clinical review was needed after a telephone consultation; however, four patients were assessed in person for reasons including pregnancy, abnormal blood results and genital herpes. Overall 90% of patients scored their satisfaction as "very high".

## Conclusions

According to standards set at inception the clinics can be considered a success with less than 1% patient dissatisfaction, greater than 90% successfully conducted telephone appointments and only one respondent indicating a preference to return to face-to-face clinics. Documentation of consent and compliance with inclusion criteria needs to be improved. However, overall telephone clinics have proven to be a convenient and effective alternative to face-to-face appointments for patients with stable HIV infection.

